# Supplementary material for: Lower Limb Deformity in Different Types of Rickets
Source: J Clin Med. 2025 Dec 4;14(23):8586. doi: 10.3390/jcm14238586 (PMC12692988; doi:10.3390/jcm14238586)
Supplement: Supplementary file 1 [file jcm-14-08586-s001.zip › jcm-3931627-supplementary.pdf]

Table S1. PRISMA 2020 Checklist.

Section and Topic	Item #	Checklist item	Location where item is reported
**TITLE**			
Title	1	Identify the report as a systematic review.	Title, p.1
**ABSTRACT**			
Abstract	2	See the PRISMA 2020 for Abstracts checklist.	Abstract, p.1
**INTRODUCTION**			
Rationale	3	Describe the rationale for the review in the context of existing knowledge.	Introduction, p. 2–3
Objectives	4	Provide an explicit statement of the objective(s) or question(s) the review addresses.	Introduction, p. 3
**METHODS**			
Eligibility criteria	5	Specify the inclusion and exclusion criteria for the review and how studies were grouped for the syntheses.	Methods p.4
Information sources	6	Specify all databases, registers, websites, organisations, reference lists and other sources searched or consulted to identify studies. Specify the date when each source was last searched or consulted.	Methods p.4
Search strategy	7	Present the full search strategies for all databases, registers and websites, including any filters and limits used.	Methods p.4
Selection process	8	Specify the methods used to decide whether a study met the inclusion criteria of the review, including how many reviewers screened each record and each report retrieved, whether they worked independently, and if applicable, details of automation tools used in the process.	Methods p.4
Data collection process	9	Specify the methods used to collect data from reports, including how many reviewers collected data from each report, whether they worked independently, any processes for obtaining or confirming data from study investigators, and if applicable, details of automation tools used in the process.	Methods p.4
Data items	10a	List and define all outcomes for which data were sought. Specify whether all results that were compatible with each outcome domain in each study were sought (e.g. for all measures, time points, analyses), and if not, the methods used to decide which results to collect.	Methods p.4
	10b	List and define all other variables for which data were sought (e.g. participant and intervention characteristics, funding sources). Describe any assumptions made about any missing or unclear information.	Methods p.4
Study risk of bias assessment	11	Specify the methods used to assess risk of bias in the included studies, including details of the tool(s) used, how many reviewers assessed each study and whether they worked independently, and if applicable, details of automation tools used in the process.	Methods “Quality and Risk of Bias Assessment” p.4
Effect measures	12	Specify for each outcome the effect measure(s) (e.g. risk ratio, mean difference) used in the synthesis or presentation of results.	n/a (descriptive only, stated in

Section and Topic	Item #	Checklist item	Location where item is reported
			Statistics, Methods p.4
Synthesis methods	13a	Describe the processes used to decide which studies were eligible for each synthesis (e.g. tabulating the study intervention characteristics and comparing against the planned groups for each synthesis (item #5)).	Methods p.4
	13b	Describe any methods required to prepare the data for presentation or synthesis, such as handling of missing summary statistics, or data conversions.	Methods p.4
	13c	Describe any methods used to tabulate or visually display results of individual studies and syntheses.	Methods p.4
	13d	Describe any methods used to synthesize results and provide a rationale for the choice(s). If meta-analysis was performed, describe the model(s), method(s) to identify the presence and extent of statistical heterogeneity, and software package(s) used.	Methods p.4
	13e	Describe any methods used to explore possible causes of heterogeneity among study results (e.g. subgroup analysis, meta-regression).	Methods p.4
	13f	Describe any sensitivity analyses conducted to assess robustness of the synthesized results.	Methods p. 5
Reporting bias assessment	14	Describe any methods used to assess risk of bias due to missing results in a synthesis (arising from reporting biases).	Methods p.4
Certainty assessment	15	Describe any methods used to assess certainty (or confidence) in the body of evidence for an outcome.	Not performed – no formal certainty assessment due to heterogeneity.
**RESULTS**			
Study selection	16a	Describe the results of the search and selection process, from the number of records identified in the search to the number of studies included in the review, ideally using a flow diagram.	Results p. 5
	16b	Cite studies that might appear to meet the inclusion criteria, but which were excluded, and explain why they were excluded.	n/a, all studies meeting inclusion criteria were included
Study characteristics	17	Cite each included study and present its characteristics.	Results, “Study characteristics”, p. 5–6; Supplementary Tables A1–A2
Risk of bias in	18	Present assessments of risk of bias for each included study.	Methods,

Section and Topic	Item #	Checklist item	Location where item is reported
studies			"Quality and Risk of Bias Assessment", p. 4; Discussion, p. 11
Results of individual studies	19	For all outcomes, present, for each study: (a) summary statistics for each group (where appropriate) and (b) an effect estimate and its precision (e.g. confidence/credible interval), ideally using structured tables or plots.	Results, p. 5–9; Tables A1–2; Supplementary Tables
Results of syntheses	20a	For each synthesis, briefly summarise the characteristics and risk of bias among contributing studies.	N/a, not risk of bias assessment performed
	20b	Present results of all statistical syntheses conducted. If meta-analysis was done, present for each the summary estimate and its precision (e.g. confidence/credible interval) and measures of statistical heterogeneity. If comparing groups, describe the direction of the effect.	Not applicable (no meta-analysis performed)
	20c	Present results of all investigations of possible causes of heterogeneity among study results.	Results p.5-9
	20d	Present results of all sensitivity analyses conducted to assess the robustness of the synthesized results.	Not performed
Reporting biases	21	Present assessments of risk of bias due to missing results (arising from reporting biases) for each synthesis assessed.	N/a
Certainty of evidence	22	Present assessments of certainty (or confidence) in the body of evidence for each outcome assessed.	Not assessed
**DISCUSSION**			
Discussion	23a	Provide a general interpretation of the results in the context of other evidence.	Discussion p.9 -12
	23b	Discuss any limitations of the evidence included in the review.	Discussion, p.11
	23c	Discuss any limitations of the review processes used.	Discussion, p.11
	23d	Discuss implications of the results for practice, policy, and future research.	Discussion, p.11
**OTHER INFORMATION**			

Section and Topic	Item #	Checklist item	Location where item is reported
Registration and protocol	24a	Provide registration information for the review, including register name and registration number, or state that the review was not registered.	Not registered, Methods p. 4
	24b	Indicate where the review protocol can be accessed, or state that a protocol was not prepared.	Methods p. 4
	24c	Describe and explain any amendments to information provided at registration or in the protocol.	Methods p. 4
Support	25	Describe sources of financial or non-financial support for the review, and the role of the funders or sponsors in the review.	Funding p.12
Competing interests	26	Declare any competing interests of review authors.	Conflict of interest p.12
Availability of data, code and other materials	27	Report which of the following are publicly available and where they can be found: template data collection forms; data extracted from included studies; data used for all analyses; analytic code; any other materials used in the review.	Appendix A

From: Page MJ, McKenzie JE, Bossuyt PM, Boutron I, Hoffmann TC, Mulrow CD, et al. The PRISMA 2020 statement: an updated guideline for reporting systematic reviews. BMJ 2021;372:n71. doi: 10.1136/bmj.n71. This work is licensed under CC BY 4.0. To view a copy of this license, visit https://creativecommons.org/licenses/by/4.0/.

Table S2. Study quality criteria.

Author	Year	N of Total Patients	N of Patients with Deformity	Definition Genu Varum (y/n)	Method Measurement Varus *	Definition Genu Valgum (y/n)	Method Measurement Valgus *	Definition Torsional Deformity (y/n)	Method Torsional Measurement *	Sagittal Deformity Measurement *	Definition Sag. Deformity (y/n)	Lab Ca (all/some/none)	Lab Phos (all/some/none)	ALP (all/some/none)
Ahmed et al. [1]	2020	62	62	n	not defined	n	not defined	n	not defined	not defined	n	mean	mean	mean
Bachrach et al. [2]	1979	24	4	n	not defined	n	not defined	n	not defined	not defined	n	all	all	all
Laditan [3]	1983	30	21	y	clinical	n	not defined	n	not defined	not defined	n	mean	mean	mean
Pettifor et al. [4]	1979	761	101	y	clinical	y	clinical	n	not defined	not defined	n	mean	mean	mean
Schnitzler et al. [5]	2019	26	26	n	not defined	n	not defined	n	not defined	not defined	n	some	some	some
Aung et al. [6]	2021	29	29	n	not defined	n	not defined	n	not defined	not defined	n	median	median	median
Blok et al. [7]	2000	18	4	n	not defined	n	not defined	n	not defined	not defined	n	all	all	all
Garabédian et al. [8]	1983	20	4	n	not defined	n	not defined	n	not defined	not defined	n	mean	mean	mean
Arnaud et al. [9]	2007	49	49	y	clinical	y	clinical	n	not defined	not defined	n	none	none	none

Hazzazi et al. [10]	2013	57	10	n	not defined	n	not defined	n	not defined	not defined	n	none	none	none
Hughes et al. [11]	2017	49	2	y	radio	y	radio	y	clinical	not defined	n	none	none	none
Wesselsky et al. [12]	2016	27	27	n	radio	n	radio	n	radio	radio	n	none	none	none
Agarwal et al. [13]	2009	51	19	y	clinical	y	clinical	n	not defined	not defined	n	mean	mean	mean
Agaja et al. [14]	2001	44	34	n	not defined	n	not defined	n	not defined	not defined	n	none	none	none
Prentice et al. [15]	2008	46	37	n	clinical	n	clinical	n	not defined	not defined	n	mean	mean	mean
El-Sobky et al. [16]	2020	50	50	y	radio	y	radio	y	radio	not defined	n	none	none	none
Wheeler et al. [17]	2015	58	14	n	not defined	n	not defined	n	not defined	not defined	n	some	some	median
Thacher et al. [18]	2013	17	8	n	not defined	n	not defined	n	not defined	not defined	n	median	median	median
Braithwaite et al. [19]	2012	4	4	n	not defined	n	not defined	n	not defined	not defined	n	none	all	none
Prakash et al. [20]	2017	117	117	y	radio	y	radio and clinical	n	not defined	not defined	n	none	none	none
DeLucia et al. [21]	2003	43	3	n	not defined	n	not defined	n	not defined	not defined	n	all	all	all
Fischer et al. [22]	1999	14	14	n	not defined	n	not defined	n	not defined	not defined	n	mean	mean	mean
Binet et al. [23]	1996	17	14	n	not defined	n	not defined	n	not defined	not defined	n	mean	mean	mean
Braithwait et al. [24]	2014	64	20	n	not defined	n	not defined	n	not defined	not defined	n	mean	mean	mean
Siddiqui et al. [25]	2005	60	5	n	not defined	n	not defined	n	not defined	not defined	n	none	none	none
Uush et al. [26]	2013	706	111	n	not defined	n	not defined	n	not defined	not defined	n	none	none	none
Chabra et al. [27]	2016	16,274	42	n	not defined	n	not defined	n	not defined	not defined	n	mean	mean	mean
Ford et al. [28]	1972	28	2	n	not defined	n	not defined	n	not defined	not defined	n	all	all	all
Pettifor et al. [29]	1978	9	8	n	not defined	n	not defined	n	not defined	not defined	n	all	all	all
Laditan et al. [30]	1975	20	15	n	not defined	n	not defined	n	not defined	not defined	n	mean	mean	mean
Narchi et al. [31]	2001	21	2	n	not defined	n	not defined	n	not defined	not defined	n	mean	mean	mean

Braithwaite et al. [32]	2016	20	20	n	not defined	n	not defined	n	not defined	not defined	n	mean	mean	mean
Thacher et al. [33]	2002	568	546	n	not defined	n	not defined	n	not defined	not defined	n	none	none	none
Takeda et al. [34]	1987	3	3	n	not defined	n	not defined	n	not defined	not defined	n	all	all	all
van der Eerden et al. [35]	2014	2	2	n	not defined	n	not defined	n	not defined	not defined	n	all	all	all
Hughes et al. [11]	2017	49	10	y	radio	y	radio	y	clinical	not defined	n	none	none	none
Méaux et al. [36]	2023	24	13	n	not defined	n	not defined	n	not defined	not defined	n	all	all	all
Faiyaz-Ul-Haque et al. [37]	2018	8	5	n	not defined	n	not defined	n	not defined	not defined	n	all	all	all
Papadopoulou et al. [38]	2014	2	2	n	not defined	n	not defined	n	not defined	not defined	n	all	all	all
Chi et al. [39]	2019	7	7	n	not defined	n	not defined	n	not defined	not defined	n	all	all	all
Wang et al. [40]	2002	6	2	n	not defined	n	not defined	n	not defined	not defined	n	all	all	some
Zargar et al. [41]	2000	3	3	n	not defined	n	not defined	n	not defined	not defined	n	all	all	all
Giraldo et al. [42]	1995	64	64	n	not defined	n	not defined	n	not defined	not defined	n	mean	mean	mean
Donghi et al. [43]	2011	2	2	n	not defined	n	not defined	n	not defined	not defined	n	all	all	all
Gribaa et al. [44]	2010	4	3	n	not defined	n	not defined	n	not defined	not defined	n	all	all	all
Econs et al. [45]	1997	23	9	n	not defined	n	not defined	n	not defined	not defined	n	some	mean	mean
Liu et al. [46]	2019	20	4	n	not defined	n	not defined	n	not defined	not defined	n	all	all	all
Gigliotti et al. [47]	1971	3	2	n	not defined	n	not defined	n	not defined	not defined	n	all	all	all
Koshida et al. [48]	2010	2	2	n	not defined	n	not defined	n	not defined	not defined	n	all	all	none
Kotwal et al. [49]	2020	9	2	n	not defined	n	not defined	n	not defined	not defined	n	all	all	all
Ni et al. [50]	2020	5	5	n	not defined	n	not defined	n	not defined	not defined	n	all	all	all
Steichen-Gersdorf et al. [51]	2015	2	2	n	clinical	n	clinical	n	not defined	not defined	n	none	all	all
Perry et al. [52]	1978	2	2	n	not defined	n	not defined	n	not defined	not defined	n	all	all	all
Mäkitie et al. [53]	2010	4	2	n	not defined	n	not defined	n	not defined	not defined	n	all	all	all
Yamamoto et al. [54]	2007	2	2	n	not defined	n	not defined	n	not defined	not defined	n	all	all	all
Yu et al. [55]	2012	12	2	n	not defined	n	clinical	n	not defined	not defined	n	all	all	all

Braithwaite et al. [56]	2013	3	3	n	not defined	n	not defined	n	not defined	not defined	n	all	all	all
Bergwitz et al. [57]	2006		4	n	not defined	n	not defined	n	not defined	not defined	n	some	all	some
Eralp et al. [58]	2004	2	2	n	not defined	n	not defined	n	not defined	not defined	n	none	none	none
Veilleux et al. [59]	2012	34	23	y	clinical	y	clinical	n	not defined	not defined	n	none	none	none
Enderle et al. [60]	1988	6	3	n	not defined	n	not defined	n	not defined	not defined	n	none	none	none
Michałus et al. [61]	2022	12	12	n	clinical	n	not defined	n	not defined	not defined	n	mean	mean	mean
Kruse et al. [62]	1998	8	5	n	not defined	n	clinical	n	not defined	not defined	n	all	all	all
Sawamura et al. [63]	2022	5	5	n	radio	n	not defined	n	not defined	radio	n	none	all	all
Jiménez et al. [64]	2021	26	25	n	not defined	n	not defined	n	not defined	not defined	n	mean	mean	mean
Berndt et al. [65]	1996	23	23	n	not defined	n	not defined	n	not defined	not defined	n	mean	mean	mean
Bonnet-Lebrun et al. [66]	2023	41	n.a.	y	radio	y	radio	y	gait	gait	n	none	mean	mean
Vakharia et al. [67]	2018	2	2	n	not defined	n	not defined	n	not defined	not defined	n	all	all	all
Song et al. [68]	2006	20	20	y	radio	y	radio	n	radio	not defined	n	none	none	none
Matsubara et al. [69]	2008	3	3	n	not defined	n	not defined	n	not defined	not defined	n	none	all	none
Petje et al. [70]	2008	10	10	y	radio	y	radio	y	radio	not defined	n	mean	mean	mean
Choi et al. [71]	2002	14	14	y	radio	y	radio	n	not defined	not defined	n	all	all	all
Mindler et al. [72]	2020	12	12	y	radio	y	radio	y	gait	not defined	n	mean	mean	mean
Bistritzer et al. [73]	1990	5	4	n	not defined	n	not defined	n	not defined	not defined	n	all	all	all
Schimert et al. [74]	1983	3	3	n	not defined	n	not defined	n	not defined	not defined	n	all	all	all
Jurca et al. [75]	2022	2	2	n	not defined	n	not defined	n	not defined	not defined	n	all	all	all
Friedman et al[76].	1993	12	7	n	not defined	n	not defined	n	not defined	not defined	n	mean	mean	none

Ekpebegh et al. [77]	2010	3	3	n	not defined	n	not defined	n	not defined	not defined	n	all	all	all
Lubani et al. [78]	1990	24	20	n	not defined	n	not defined	n	not defined	not defined	n	all	all	all
Zobel et al. [79]	1990	2	2	n	not defined	n	not defined	n	not defined	not defined	n	all	all	all
Zhao et al. [80]	2020	3	3	n	not defined	n	not defined	n	not defined	not defined	n	all	all	all
Ziller[81]	1977	2	2	y	clinical	n	not defined	n	not defined	not defined	n	none	none	none
McNair et al. [82]	1969	36	16	n	not defined	n	not defined	n	not defined	not defined	n	none	none	none
Bhadada et al. [83]	2010	17	12	n	not defined	n	not defined	n	not defined	not defined	n	mean	mean	mean
Novais et al. [84]	2006	10	10	n	radio	n	radio	n	not defined	not defined	n	none	none	none
Yuan et al. [85]	2015	4	4	n	not defined	n	not defined	n	not defined	not defined	n	all	all	all
Cao et al. [86]	2022	17	8	n	not defined	n	not defined	n	not defined	not defined	n	mean	mean	mean
Mindler et al. [87]	2021	43	43	y	radio	y	radio	y	not defined	gait	y	mean	mean	mean
Lempicki et al. [88]	2017	27	20	n	clinical	n	clinical	n	not defined	not defined	n	none	none	mean
Fucentese et al. [89]	2008	12	7	n	not defined	n	not defined	n	not defined	not defined	n	all	all	none
Makitie et al. [90]	2008	10	6	n	not defined	n	not defined	n	not defined	not defined	n	mean	mean	mean
Rush[91]	2022	519	190	n	not defined	n	not defined	n	not defined	not defined	n	none	none	none
Uday et al [92].	2021	38	20	n	not defined	n	not defined	n	not defined	not defined	n	none	none	mean
Şıklar et al. [93]	2020	166	146	n	clinical	n	clinical	n	not defined	not defined	n	mean	mean	mean
Huang et al. [94]	2015	7	7	n	not defined	n	not defined	n	not defined	not defined	n	some	some	some
Chou et al. [95]	2005	2	2	n	not defined	n	not defined	n	not defined	not defined	n	all	all	all
Kienitz et al. [96]	2011	3	2	n	not defined	n	not defined	n	not defined	not defined	n	all	all	none
Eyres et al. [97]	1993	7	7	n	not defined	n	not defined	n	not defined	not defined	n	none	none	none
Gizard et al. [98]	2017	49	49	n	clinical	n	clinical	n	not defined	not defined	n	none	none	none
Mindler et al. [99]	2022	7	7	y	radio	y	radio	y	radio	not defined	n	none	mean	mean
Grote et al. [100]	2023	24	24	y	radio	y	radio	n	not defined	not defined	n	none	none	none
Sandy et al. [101]	2023	93	81	n	not defined	n	not defined	n	not defined	not defined	n	mean	mean	mean
Moreira et al. [102]	2021	19	17	n	not defined	n	not defined	n	not defined	not defined	n	median	median	median
Baroncelli et al. [103]	2006	9	2	n	clinical	n	not defined	n	not defined	not defined	n	none	all	all

Evans et al. [104]	1980	10	4	n	not defined	n	not defined	n	not defined	not defined	n	mean	mean	mean
Rubinovitch et al. [105]	1988	10	10	n	not defined	n	not defined	n	not defined	not defined	n	none	none	none
Bonnet-Lebrun et al. [106]	2023	35	35	y	radio	y	radio	n	radio	not defined	n	none	mean	mean
Aiello et al. [107]	2023	4	4	y	clinical	n	not defined	n	not defined	not defined	n	some	some	some
Popkov et al. [108]	2015	47	47	n	radio	n	radio	n	not defined	not defined	n	none	none	none
Greene et al. [109]	1985	8	8	n	not defined	n	not defined	n	not defined	not defined	n	none	none	none
Feng et al. [110]	2023	26	26	y	radio	y	radio	n	not defined	not defined	n	none	none	none
Loeffler et al. [111]	1982	13	13	n	not defined	n	not defined	n	not defined	not defined	n	all	all	all
Horn et al. [112]	2017	24	24	y	radio	y	radio	n	not defined	clinical	n	none	mean	mean
Song et al. [113]	2014	39	39	y	radio	y	radio	n	not defined	not defined	n	mean	mean	mean
Zagari et al. [114]	2022	2	2	n	not defined	n	not defined	n	not defined	not defined	n	all	all	all
McAlister et al. [115]	1987	2	2	n	not defined	n	not defined	n	not defined	not defined	n	none	none	none
Eguchi et al. [116]	1980	16	3	n	not defined	n	not defined	n	not defined	not defined	n	all	all	all
Kanel et al. [117]	1995	9	9	n	not defined	n	not defined	n	not defined	not defined	n	none	none	none
Litman et al. [118]	1957	10	8	n	not defined	n	not defined	n	not defined	not defined	n	mean	mean	mean
Godfrey et al. [119]	2020	2	2	n	not defined	n	not defined	n	not defined	not defined	n	all	all	all
Tavana et al. [120]	2022	3	3	n	not defined	n	not defined	n	not defined	not defined	n	all	all	all
Al Kaissi et al. [121]	2013	7	7	n	not defined	n	not defined	n	not defined	not defined	n	none	none	none
Dahir et al. [122]	2022	22	9	n	not defined	n	not defined	n	not defined	not defined	n	some	some	some
Reid et al. [123]	1989	22	18	n	clinical	n	clinical	n	not defined	not defined	n	all	all	all
Smith et al. [124]	2020	30	12	n	not defined	n	not defined	n	not defined	not defined	n	none	mean	none
Emma et al. [125]	2019	175	166	n	not defined	n	not defined	n	not defined	not defined	n	none	none	none
Nielsen et al. [126]	2014	15	7	n	clinical	n	clinical	n	not defined	not defined	n	none	none	none

Calcium deficiency rickets VDDR ADHR ARHR HHRH HPR/XLH. * (radio/clinical/gait/not defined).

Table S3. Deformity description.

Author	Year	Deformity/Bowing	Genu Varus	Genu Valgus	Torsional Deformity	Torsion Femur	Torsion Tibia	Sagittal Deformity Femur (Prokurvatum)	Sagittal Deformity Femur (Recurvatum)	Sagittal Deformity Tibia (Procurvatum/Recurvatum)	Windswept Deformity Patients
Ahmed et al. [1]	2020		70	54							
Bachrach et al. [2]	1979	2		2	4						
Laditan[3]	1983		42								
Pettifor et al. [4]	1979		140	62							
Schnitzler et al. [5]	2019	52									
Aung et al. [6]	2021		16	42							
Blok et al. [7]	2000	4									
Garabédian et al. [8]	1983	4									
Arnaud et al. [9]	2007		50	46							2
Hazzazi et al. [10]	2013	20									
Hughes et al. [11]	2017		0	2							
Wesselsky et al. [12]	2016		15	28	5					7	
Agarwal et al. [13]	2009	19	30	8	0						
Agaja et al. [14]	2001	68									
Prentice et al. [15]	2008		54	18							1
El-Sobky et al. [16]	2020		51	35							
Wheeler et al. [17]	2015	28									
Thacher et al. [18]	2013	16									
Braithwaite et al. [19]	2012	8									
Prakash et al. [20]	2017		60	128							5
DeLucia et al. [21]	2003	6					4				
Fischer et al. [22]	1999	8		20							
Binet et al. [23]	1996	28					2				
Braithwait et al. [24]	2014	40									
Siddiqui et al. [25]	2005	10									
Uush et al. [26]	2013	222									
Chabra et al. [27]	2016	26	28	48							1

Ford et al. [28]	1972			4				
Pettifor et al. [29]	1978	14						1
Laditan et al. [30]	1975	30						
Narchi et al. [31]	2001	4						
Braithwaite et al. [32]	2016	40						
Thacher et al. [33]	2002		454	280				76
Takeda et al. [34]	1987		0	6		x		
van der Eerden et al. [35]	2014		4					
Hughes et al. [11]	2017		6	4		3		
Méaux et al. [36]	2023	2	14	10				
Faiyaz-Ul-Haque et al. [37]	2018	8		2				
Papadopoulou et al. [38]	2014	2						1
Chi et al. [39]	2019		10	4				
Wang et al. [40]	2002	2	2					
Zargar et al. [41]	2000	2		4				
Giraldo et al. [42]	1995		27	32				
Donghi et al. [43]	2011		2	1			2	
Gribaa et al. [44]	2010	6						
Econs et al. [45]	1997	16						1
Liu et al. [46]	2019	2	1	1	0			
Gigliotti et al. [47]	1971	3						
Koshida et al. [48]	2010		4					
Kotwal et al. [49]	2020	4						
Ni et al. [50]	2020	2	4	4				
Steichen-Gersdorf et al. [51]	2015		4	4				
Perry et al. [52]	1978	4						
Mäkitie et al. [53]	2010		4					
Yamamoto et al. [54]	2007	4						
Yu et al. [55]	2012			4				
Braithwaite et al. [56]	2013	4		2				
Bergwitz et al. [57]	2006	2	2	4				
Eralp et al. [58]	2004							2

Veilleux et al. [59]	2012	4	18	24						
Enderle et al. [60]	1988		4	2						
Michałus et al. [61]	2022		24							
Kruse et al. [62]	1998	6	2	2						
Sawamura et al. [63]	2022		10				10		10	
Jiménez et al. [64]	2021	44								
Berndt et al. [65]	1996		5	26				6		
Bonnet-Lebrun et al. [66]	2023		36	20		40				
Vakharia et al. [67]	2018	4								
Song et al. [68]	2006		11	7	0					2
Matsubara et al. [69]	2008	6			0					
Petje et al. [70]	2008		19	16	1					
Choi et al. [71]	2002		20	8						
Mindler et al. [72]	2020		11	7	12					
Bistritzer et al. [73]	1990	8								
Schimert et al. [74]	1983	6								
Jurca et al. [75]	2022		2							1
Friedman et al[76].	1993	14								
Ekpebegh et al. [77]	2010	2	2							1
Lubani et al. [78]	1990	40								
Zobel et al. [79]	1990	4								
Zhao et al. [80]	2020	6								
Ziller[81]	1977		4							
McNair et al. [82]	1969	32								
Bhadada et al. [83]	2010		18	4						1
Novais et al. [84]	2006		7	12						
Yuan et al. [85]	2015	8								
Cao et al. [86]	2022	8	2	6						
Mindler et al. [87]	2021		39	6			32		42	
Lempicki et al. [88]	2017		26	14						
Fucentese et al. [89]	2008	2	6	6						
Makitie et al. [90]	2008		12							
Rush[91]	2022	190								
Uday et al. [92]	2021	40								

Şıklar et al. [93]	2020		266	26						
Huang et al. [94]	2015		14							
Chou et al. [95]	2005		4							
Kienitz et al. [96]	2011		4							
Eyres et al. [97]	1993	11		3						
Gizard et al. [98]	2017		64	34						
Mindler et al. [99]	2022		3	5	8	9				
Grote et al. [100]	2023		36	12						
Sandy et al. [101]	2023	162								
Moreira et al. [102]	2021	34								
Baroncelli et al. [103]	2006	2	2							
Evans et al. [104]	1980		5							1
Rubinovitch et al. [105]	1988	20								
Bonnet-Lebrun et al. [106]	2023		23	25	19	28				
Aiello et al. [107]	2023		8							
Popkov et al. [108]	2015		86	8			43		43	
Greene et al. [109]	1985	16								
Feng et al. [110]	2023		31	21						
Loeffler et al. [111]	1982		22	4						
Horn et al. [112]	2017		18	12			0	0		
Song et al. [113]	2014		58	8						
Zagari et al. [114]	2022		4							
McAlister et al. [115]	1987		4							
Eguchi et al. [116]	1980	6								
Kanel et al. [117]	1995	18								
Litman et al. [118]	1957	16								
Godfrey et al. [119]	2020			4						
Tavana et al. [120]	2022	6								
Al Kaissi et al. [121]	2013									7
Dahir et al. [122]	2022	18								
Reid et al. [123]	1989		32	2						1
Smith et al. [124]	2020	12		12		2				
Emma et al. [125]	2019	332								
Nielsen et al. [126]	2014		10	4						

Calcium deficiency rickets VDDR ADHR ARHR HHRH HPR/XLH.

References

1. Ahmed, S.; Goldberg, G.R.; Raqib, R.; Roy, S.K.; Haque, S.; Braithwaite, V.S.; Pettifor, J.M.; Prentice, A. Aetiology of Nutritional Rickets in Rural Bangladeshi Children. *Bone* **2020**, *136*, 115357, doi:10.1016/j.bone.2020.115357.
2. Bachrach, S.; Fisher, J.; Parks, J.S. An Outbreak of Vitamin D Deficiency Rickets in a Susceptible Population. *Pediatrics* **1979**, *64*, 871–877.
3. Laditan, A.A. Bilateral Genu Vara in Childhood. *Cent Afr J Med* **1983**, *29*, 219–223.
4. Pettifor, J.M.; Ross, P.; Moodley, G.; Shuenyane, E. Calcium Deficiency in Rural Black Children in South Africa--a Comparison between Rural and Urban Communities. *Am J Clin Nutr* **1979**, *32*, 2477–2483, doi:10.1093/ajcn/32.12.2477.
5. Schnitzler, C.M.; Pettifor, J.M. Calcium Deficiency Rickets in African Adolescents: Cortical Bone Histomorphometry. *JBMR Plus* **2019**, *3*, e10169, doi:10.1002/jbm4.10169.
6. Aung, H.; Soe, K.; Smithuis, F.F.; Lamb, T.; Aung, M.W.; Smithuis, F.M. Case Report: Children with Severe Nutritional Rickets in the Naga Region in Northwest Myanmar, on the Border with India. *Am J Trop Med Hyg* **2021**, *105*, 217–221, doi:10.4269/ajtmh.20-1431.
7. Blok, B.H.; Grant, C.C.; McNeil, A.R.; Reid, I.R. Characteristics of Children with Florid Vitamin D Deficient Rickets in the Auckland Region in 1998. *N Z Med J* **2000**, *113*, 374–376.
8. Garabédian, M.; Vainsel, M.; Mallet, E.; Guillozo, H.; Toppet, M.; Grimberg, R.; NGuyen, T.M.; Balsan, S. Circulating Vitamin D Metabolite Concentrations in Children with Nutritional Rickets. *J Pediatr* **1983**, *103*, 381–386, doi:10.1016/s0022-3476(83)80407-7.
9. Arnaud, J.; Pettifor, J.M.; Cimma, J.P.; Fischer, P.R.; Craviari, T.; Meisner, C.; Haque, S. Clinical and Radiographic Improvement of Rickets in Bangladeshi Children as a Result of Nutritional Advice. *Ann Trop Paediatr* **2007**, *27*, 185–191, doi:10.1179/146532807X220299.
10. Hazzazi, M.A.; Alzeer, I.; Tamimi, W.; Al Atawi, M.; Al Alwan, I. Clinical Presentation and Etiology of Osteomalacia/Rickets in Adolescents. *Saudi J Kidney Dis Transpl* **2013**, *24*, 938–941, doi:10.4103/1319-2442.118087.
11. Hughes, A.; Heidari, N.; Mitchell, S.; Livingstone, J.; Jackson, M.; Atkins, R.; Monsell, F. Computer Hexapod-Assisted Orthopaedic Surgery Provides a Predictable and Safe Method of Femoral Deformity Correction. *Bone Joint J* **2017**, *99-B*, 283–288, doi:10.1302/0301-620X.99B2.BJJ-2016-0271.R1.
12. Wesselsky, V.; Kitz, C.; Jakob, F.; Eulert, J.; Raab, P. Description and Evaluation of Operative Deformity Correction in Calcium-Deficiency Rickets in Kaduna, Northern Nigeria. *Int Orthop* **2016**, *40*, 653–658, doi:10.1007/s00264-015-2911-7.
13. Agarwal, A.; Gulati, D. Early Adolescent Nutritional Rickets. *J Orthop Surg (Hong Kong)* **2009**, *17*, 340–345, doi:10.1177/230949900901700320.
14. Agaja, S.B. Factors Affecting Angular Deformities of the Knees in Nigerian Children--Ilorin Experience. *West Afr J Med* **2001**, *20*, 246–250.
15. Prentice, A.; Ceesay, M.; Nigdikar, S.; Allen, S.J.; Pettifor, J.M. FGF23 Is Elevated in Gambian Children with Rickets. *Bone* **2008**, *42*, 788–797, doi:10.1016/j.bone.2007.11.014.
16. El-Sobky, T.A.; Samir, S.; Baraka, M.M.; Fayyad, T.A.; Mahran, M.A.; Aly, A.S.; Amen, J.; Mahmoud, S. Growth Modulation for Knee Coronal Plane Deformities in Children With Nutritional Rickets: A Prospective Series With Treatment Algorithm. *J Am Acad Orthop Surg Glob Res Rev* **2020**, *4*, doi:10.5435/JAAOSGlobal-D-19-00009.
17. Wheeler, B.J.; Dickson, N.P.; Houghton, L.A.; Ward, L.M.; Taylor, B.J. Incidence and Characteristics of Vitamin D Deficiency Rickets in New Zealand Children: A New Zealand Paediatric Surveillance Unit Study. *Aust N Z J Public Health* **2015**, *39*, 380–383, doi:10.1111/1753-6405.12390.
18. Thacher, T.D.; Fischer, P.R.; Tebben, P.J.; Singh, R.J.; Cha, S.S.; Maxson, J.A.; Yawn, B.P. Increasing Incidence of Nutritional Rickets: A Population-Based Study in Olmsted County, Minnesota. *Mayo Clin Proc* **2013**, *88*, 176–183, doi:10.1016/j.mayocp.2012.10.018.
19. Braithwaite, V.; Bruggraber, S.F.A.; Prentice, A. Intact Fibroblast Growth Factor 23 and Fragments in Plasma from Gambian Children. *Osteoporos Int* **2013**, *24*, 1121–1124, doi:10.1007/s00198-012-2029-3.

20. Prakash, J.; Mehtani, A.; Sud, A.; Reddy, B.K. Is Surgery Always Indicated in Rachitic Coronal Knee Deformities? Our Experience in 198 Knees. *J Orthop Surg (Hong Kong)* **2017**, *25*, 2309499017693532, doi:10.1177/2309499017693532.
21. DeLucia, M.C.; Mitnick, M.E.; Carpenter, T.O. Nutritional Rickets with Normal Circulating 25-Hydroxyvitamin D: A Call for Reexamining the Role of Dietary Calcium Intake in North American Infants. *J Clin Endocrinol Metab* **2003**, *88*, 3539–3545, doi:10.1210/jc.2002-021935.
22. Fischer, P.R.; Rahman, A.; Cimma, J.P.; Kyaw-Myint, T.O.; Kabir, A.R.; Talukder, K.; Hassan, N.; Manaster, B.J.; Staab, D.B.; Duxbury, J.M.; et al. Nutritional Rickets without Vitamin D Deficiency in Bangladesh. *J Trop Pediatr* **1999**, *45*, 291–293, doi:10.1093/tropej/45.5.291.
23. Binet, A.; Kooh, S.W. Persistence of Vitamin D-Deficiency Rickets in Toronto in the 1990s. *Can J Public Health* **1996**, *87*, 227–230.
24. Braithwaite, V.; Pettifor, J.M.; Prentice, A. Novel SLC34A3 Mutation Causing Hereditary Hypophosphataemic Rickets with Hypercalciuria in a Gambian Family. *Bone* **2013**, *53*, 216–220, doi:10.1016/j.bone.2012.12.003.
25. Siddiqui, T.S.; Rai, M.I. Presentation and Predisposing Factors of Nutritional Rickets in Children of Hazara Division. *J Ayub Med Coll Abbottabad* **2005**, *17*, 29–32.
26. Uush, T. Prevalence of Classic Signs and Symptoms of Rickets and Vitamin D Deficiency in Mongolian Children and Women. *J Steroid Biochem Mol Biol* **2013**, *136*, 207–210, doi:10.1016/j.jsbmb.2012.10.014.
27. Chabra, T.; Tahbildar, P.; Sharma, A.; Boruah, S.; Mahajan, R.; Raje, A. Prevalence of Skeletal Deformity Due to Nutritional Rickets in Children between 1 and 18 Years in Tea Garden Community. *J Clin Orthop Trauma* **2016**, *7*, 86–89, doi:10.1016/j.jcot.2016.01.005.
28. Ford, J.A.; Colhoun, E.M.; McIntosh, W.B.; Dunnigan, M.G. Rickets and Osteomalacia in the Glasgow Pakistani Community, 1961-71. *Br Med J* **1972**, *2*, 677–680, doi:10.1136/bmj.2.5815.677.
29. Pettifor, J.M.; Ross, P.; Wang, J.; Moodley, G.; Couper-Smith, J. Rickets in Children of Rural Origin in South Africa: Is Low Dietary Calcium a Factor? *J Pediatr* **1978**, *92*, 320–324, doi:10.1016/s0022-3476(78)80035-3.
30. Laditan, A.A.; Adeniyi, A. Rickets in Nigerian Children--Response to Vitamin D. *J Trop Med Hyg* **1975**, *78*, 206–209.
31. Narchi, H.; El Jamil, M.; Kulaylat, N. Symptomatic Rickets in Adolescence. *Arch Dis Child* **2001**, *84*, 501–503, doi:10.1136/adc.84.6.501.
32. Braithwaite, V.S.; Freeman, R.; Greenwood, C.L.; Summers, D.M.; Nigdikar, S.; Lavy, C.B.D.; Offiah, A.C.; Bishop, N.J.; Cashman, J.; Prentice, A. The Aetiology of Rickets-like Lower Limb Deformities in Malawian Children. *Osteoporos Int* **2016**, *27*, 2367–2372, doi:10.1007/s00198-016-3541-7.
33. Thacher, T.D.; Fischer, P.R.; Pettifor, J.M. The Usefulness of Clinical Features to Identify Active Rickets. *Ann Trop Paediatr* **2002**, *22*, 229–237, doi:10.1179/027249302125001525.
34. Takeda, E.; Kuroda, Y.; Saijo, T.; Naito, E.; Kobashi, H.; Yokota, I.; Miyao, M. 1 Alpha-Hydroxyvitamin D3 Treatment of Three Patients with 1,25-Dihydroxyvitamin D-Receptor-Defect Rickets and Alopecia. *Pediatrics* **1987**, *80*, 97–101.
35. van der Eerden, B.C.J.; van der Heyden, J.C.; van Hamburg, J.P.; Schreuders-Koedam, M.; Asmawidjaja, P.S.; de Muinck Keizer-Schrama, S.M.; Boot, A.M.; Lubberts, E.; Drop, S.L.S.; van Leeuwen, J.P.T.M. A Human Vitamin D Receptor Mutation Causes Rickets and Impaired Th1/Th17 Responses. *Bone* **2014**, *69*, 6–11, doi:10.1016/j.bone.2014.08.005.
36. Méaux, M.-N.; Harambat, J.; Rothenbuhler, A.; Léger, J.; Kamenicky, P.; Soskin, S.; Boyer, O.; Boros, E.; D'Anella, P.; Mignot, B.; et al. Genotype-Phenotype Description of Vitamin D-Dependent Rickets 1A: CYP27B1 p.(Ala129Thr) Variant Induces a Milder Disease. *J Clin Endocrinol Metab* **2023**, *108*, 812–826, doi:10.1210/clinem/dgac639.
37. Faiyaz-Ul-Haque, M.; AlDhalaan, W.; AlAshwal, A.; Bin-Abbas, B.S.; AlSagheir, A.; Alotaiby, M.; Rafiq, Z.; Zaidi, S.H.E. Hereditary 1,25-Dihydroxyvitamin D-Resistant Rickets (HVDRR): Clinical Heterogeneity and Long-Term Efficacious Management of Eight Patients from Four Unrelated Arab Families with a Loss of Function VDR Mutation. *J Pediatr Endocrinol Metab* **2018**, *31*, 861–868, doi:10.1515/jpem-2017-0312.
38. Papadopoulou, A.; Bountouvi, E.; Gole, E.; Doulgeraki, A.; Tournis, S.; Papadimitriou, A.; Nicolaidou, P. Identification of a Novel Nonsense Mutation in the Ligand-Binding Domain of the Vitamin d Receptor

Gene and Clinical Description of Two Greek Patients with Hereditary Vitamin d-Resistant Rickets and Alopecia. *Horm Res Paediatr* **2014**, *82*, 206–212, doi:10.1159/000362618.

39. Chi, Y.; Sun, J.; Pang, L.; Jiajue, R.; Jiang, Y.; Wang, O.; Li, M.; Xing, X.; Hu, Y.; Zhou, X.; et al. Mutation Update and Long-Term Outcome after Treatment with Active Vitamin D(3) in Chinese Patients with Pseudovitamin D-Deficiency Rickets (PDDR). *Osteoporos Int* **2019**, *30*, 481–489, doi:10.1007/s00198-018-4607-5.
40. Wang, X.; Zhang, M.Y.H.; Miller, W.L.; Portale, A.A. Novel Gene Mutations in Patients with 1alpha-Hydroxylase Deficiency That Confer Partial Enzyme Activity in Vitro. *J Clin Endocrinol Metab* **2002**, *87*, 2424–2430, doi:10.1210/jcem.87.6.8534.
41. Zargar, A.H.; Mithal, A.; Wani, A.I.; Laway, B.A.; Masoodi, S.R.; Bashir, M.I.; Ganie, M.A. Pseudovitamin D Deficiency Rickets--a Report from the Indian Subcontinent. *Postgrad Med J* **2000**, *76*, 369–372, doi:10.1136/pmj.76.896.369.
42. Giraldo, A.; Pino, W.; García-Ramírez, L.F.; Pineda, M.; Iglesias, A. Vitamin D Dependent Rickets Type II and Normal Vitamin D Receptor cDNA Sequence. A Cluster in a Rural Area of Cauca, Colombia, with More than 200 Affected Children. *Clin Genet* **1995**, *48*, 57–65, doi:10.1111/j.1399-0004.1995.tb04056.x.
43. Donghi, V.; Di Frenna, M.; di Lascio, A.; Chiumello, G.; Weber, G. Vitamin D Dependent Rickets, Diagnostic and Therapeutic Difficulties: Two Case Reports. *J Pediatr Endocrinol Metab* **2011**, *24*, 801–805, doi:10.1515/jpem.2011.214.
44. Gribaa, M.; Younes, M.; Bouyacoub, Y.; Korbaa, W.; Ben Charfeddine, I.; Touzi, M.; Adala, L.; Mamay, O.; Bergaoui, N.; Saad, A. An Autosomal Dominant Hypophosphatemic Rickets Phenotype in a Tunisian Family Caused by a New FGF23 Missense Mutation. *J Bone Miner Metab* **2010**, *28*, 111–115, doi:10.1007/s00774-009-0111-5.
45. Econs, M.J.; McEnery, P.T. Autosomal Dominant Hypophosphatemic Rickets/Osteomalacia: Clinical Characterization of a Novel Renal Phosphate-Wasting Disorder. *J Clin Endocrinol Metab* **1997**, *82*, 674–681, doi:10.1210/jcem.82.2.3765.
46. Liu, C.; Zhao, Z.; Wang, O.; Li, M.; Xing, X.; Hsieh, E.; Fukumoto, S.; Jiang, Y.; Xia, W. Earlier Onset in Autosomal Dominant Hypophosphatemic Rickets of R179 than R176 Mutations in Fibroblast Growth Factor 23: Report of 20 Chinese Cases and Review of the Literature. *Calcif Tissue Int* **2019**, *105*, 476–486, doi:10.1007/s00223-019-00597-y.
47. Gigliotti, R.; Harrison, H.; Reveley, R.A.; Drabkowski, A.J. Familial Vitamin D-Refractory Rickets. *J Am Dent Assoc* **1971**, *82*, 383–387, doi:10.14219/jada.archive.1971.0064.
48. Koshida, R.; Yamaguchi, H.; Yamasaki, K.; Tsuchimochi, W.; Yonekawa, T.; Nakazato, M. A Novel Nonsense Mutation in the DMP1 Gene in a Japanese Family with Autosomal Recessive Hypophosphatemic Rickets. *J Bone Miner Metab* **2010**, *28*, 585–590, doi:10.1007/s00774-010-0169-0.
49. Kotwal, A.; Ferrer, A.; Kumar, R.; Singh, R.J.; Murthy, V.; Schultz-Rogers, L.; Zimmermann, M.; Lanpher, B.; Zimmerman, K.; Stabach, P.R.; et al. Clinical and Biochemical Phenotypes in a Family With ENPP1 Mutations. *J Bone Miner Res* **2020**, *35*, 662–670, doi:10.1002/jbmr.3938.
50. Ni, X.; Li, X.; Zhang, Q.; Liu, C.; Gong, Y.; Wang, O.; Li, M.; Xing, X.; Jiang, Y.; Xia, W. Clinical Characteristics and Bone Features of Autosomal Recessive Hypophosphatemic Rickets Type 1 in Three Chinese Families: Report of Five Chinese Cases and Review of the Literature. *Calcif Tissue Int* **2020**, *107*, 636–648, doi:10.1007/s00223-020-00755-7.
51. Steichen-Gersdorf, E.; Lorenz-Depiereux, B.; Strom, T.M.; Shaw, N.J. Early Onset Hearing Loss in Autosomal Recessive Hypophosphatemic Rickets Caused by Loss of Function Mutation in ENPP1. *J Pediatr Endocrinol Metab* **2015**, *28*, 967–970, doi:10.1515/jpem-2014-0531.
52. Perry, W.; Stamp, T.C. Hereditary Hypophosphataemic Rickets with Autosomal Recessive Inheritance and Severe Osteosclerosis. A Report of Two Cases. *J Bone Joint Surg Br* **1978**, *60-B*, 430–434, doi:10.1302/0301-620X.60B3.681423.
53. Mäkitie, O.; Pereira, R.C.; Kaitila, I.; Turan, S.; Bastepe, M.; Laine, T.; Kröger, H.; Cole, W.G.; Jüppner, H. Long-Term Clinical Outcome and Carrier Phenotype in Autosomal Recessive Hypophosphatemia Caused by a Novel DMP1 Mutation. *J Bone Miner Res* **2010**, *25*, 2165–2174, doi:10.1002/jbmr.105.

54. Yamamoto, T.; Michigami, T.; Aranami, F.; Segawa, H.; Yoh, K.; Nakajima, S.; Miyamoto, K.; Ozono, K. Hereditary Hypophosphatemic Rickets with Hypercalciuria: A Study for the Phosphate Transporter Gene Type IIc and Osteoblastic Function. *J Bone Miner Metab* **2007**, *25*, 407–413, doi:10.1007/s00774-007-0776-6.
55. Yu, Y.; Sanderson, S.R.; Reyes, M.; Sharma, A.; Dunbar, N.; Srivastava, T.; Jüppner, H.; Bergwitz, C. Novel NaPi-IIc Mutations Causing HHRH and Idiopathic Hypercalciuria in Several Unrelated Families: Long-Term Follow-up in One Kindred. *Bone* **2012**, *50*, 1100–1106, doi:10.1016/j.bone.2012.02.015.
56. Braithwaite, V.; Jones, K.S.; Assar, S.; Schoenmakers, I.; Prentice, A. Predictors of Intact and C-Terminal Fibroblast Growth Factor 23 in Gambian Children. *Endocr Connect* **2014**, *3*, 1–10, doi:10.1530/EC-13-0070.
57. Bergwitz, C.; Roslin, N.M.; Tieder, M.; Loredo-Osti, J.C.; Bastepe, M.; Abu-Zahra, H.; Frappier, D.; Burkett, K.; Carpenter, T.O.; Anderson, D.; et al. SLC34A3 Mutations in Patients with Hereditary Hypophosphatemic Rickets with Hypercalciuria Predict a Key Role for the Sodium-Phosphate Cotransporter NaPi-IIc in Maintaining Phosphate Homeostasis. *Am J Hum Genet* **2006**, *78*, 179–192, doi:10.1086/499409.
58. Eralp, L.; Kocaoglu, M.; Cakmak, M.; Ozden, V.E. A Correction of Windswept Deformity by Fixator Assisted Nailing. A Report of Two Cases. *J Bone Joint Surg Br* **2004**, *86*, 1065–1068, doi:10.1302/0301-620x.86b7.14923.
59. Veilleux, L.-N.; Cheung, M.; Ben Amor, M.; Rauch, F. Abnormalities in Muscle Density and Muscle Function in Hypophosphatemic Rickets. *J Clin Endocrinol Metab* **2012**, *97*, E1492-1498, doi:10.1210/jc.2012-1336.
60. Enderle, A. [Biologico-mechanical behavior of bone in osteomalacia and in phosphate diabetes in adulthood]. *Orthopade* **1988**, *17*, 432–439.
61. Michałus, I.; Łupińska, A.; Woch, I.; Wieczorek-Szukała, K.; Chlebna-Sokół, D.; Lewiński, A. Bone Turnover Markers and Bone Mineral Density in Children with Hypophosphatemic Rickets. *J Clin Med* **2022**, *11*, doi:10.3390/jcm11154622.
62. Kruse, K.; Hinkel, G.K.; Griefahn, B. Calcium Metabolism and Growth during Early Treatment of Children with X-Linked Hypophosphataemic Rickets. *Eur J Pediatr* **1998**, *157*, 894–900, doi:10.1007/s004310050962.
63. Sawamura, K.; Hamajima, T.; Izawa, M.; Kaneko, H.; Kitamura, A.; Kitoh, H. Changes of the Lower Limb Deformity in Children with FGF23-Related Hypophosphatemic Rickets Treated with Burosumab: A Single-Center Prospective Study. *J Pediatr Orthop B* **2022**, doi:10.1097/BPB.0000000000001054.
64. Jiménez, M.; Ivanovic-Zuvic, D.; Loureiro, C.; Carvajal, C.A.; Cavada, G.; Schneider, P.; Gallardo, E.; García, C.; Gonzalez, G.; Contreras, O.; et al. Clinical and Molecular Characterization of Chilean Patients with X-Linked Hypophosphatemia. *Osteoporos Int* **2021**, *32*, 1825–1836, doi:10.1007/s00198-021-05875-w.
65. Berndt, M.; Ehrich, J.H.; Lazovic, D.; Zimmermann, J.; Hillmann, G.; Kayser, C.; Prokop, M.; Schirg, E.; Siegert, B.; Wolff, G.; et al. Clinical Course of Hypophosphatemic Rickets in 23 Adults. *Clin Nephrol* **1996**, *45*, 33–41.
66. Bonnet-Lebrun, A.; Linglart, A.; De Tienda, M.; Nguyen Khac, V.; Ouchrif, Y.; Berkenou, J.; Pillet, H.; Assi, A.; Wicart, P.; Skalli, W. Combined Gait Analysis and Radiologic Examination in Children with X-Linked Hypophosphatemia. *Clin. Biomech.* **2023**, *105*, 105974. https://doi.org/10.1016/j.clinbiomech.2023.105974.
67. Vakharia, J.D.; Matlock, K.; Taylor, H.O.; Backeljauw, P.F.; Topor, L.S. Craniosynostosis as the Presenting Feature of X-Linked Hypophosphatemic Rickets. *Pediatrics* **2018**, *141*, S515–S519, doi:10.1542/peds.2017-2522.
68. Song, H.-R.; Soma Raju, V.V.J.; Kumar, S.; Lee, S.-H.; Suh, S.-W.; Kim, J.-R.; Hong, J.-S. Deformity Correction by External Fixation and/or Intramedullary Nailing in Hypophosphatemic Rickets. *Acta Orthop* **2006**, *77*, 307–314, doi:10.1080/17453670610046073.
69. Matsubara, H.; Tsuchiya, H.; Kabata, T.; Sakurakichi, K.; Watanabe, K.; Tomita, K. Deformity Correction for Vitamin D-Resistant Hypophosphatemic Rickets of Adults. *Arch Orthop Trauma Surg* **2008**, *128*, 1137–1143, doi:10.1007/s00402-007-0548-8.
70. Petje, G.; Meizer, R.; Radler, C.; Aigner, N.; Grill, F. Deformity Correction in Children with Hereditary Hypophosphatemic Rickets. *Clin. Orthop. Relat. Res.* **2008**, *466*, 3078–3085. https://doi.org/10.1007/s11999-008-0547-2.

71. Choi, I.H.; Kim, J.K.; Chung, C.Y.; Cho, T.-J.; Lee, S.H.; Suh, S.W.; Whang, K.S.; Park, H.W.; Song, K.S. Deformity Correction of Knee and Leg Lengthening by Ilizarov Method in Hypophosphatemic Rickets: Outcomes and Significance of Serum Phosphate Level. *J. Pediatr. Orthop.* **2002**, *22*, 626–631.
72. Mindler, G.T.; Kranzl, A.; Stauffer, A.; Haeusler, G.; Ganger, R.; Raimann, A. Disease-Specific Gait Deviations in Pediatric Patients with X-Linked Hypophosphatemia. *Gait Posture* **2020**, *81*, 78–84. https://doi.org/10.1016/j.gaitpost.2020.07.007.
73. Bistritzer, T.; Chalew, S.A.; Hanukoglu, A.; Armour, K.M.; Haney, P.J.; Kowarski, A.A. Does Growth Hormone Influence the Severity of Phosphopenic Rickets? *Eur J Pediatr* **1990**, *150*, 26–29, doi:10.1007/BF01959474.
74. Schimert, G.; Fanconi, A. Early History of Familial Hypophosphataemic Vitamin D-Resistant Rickets. Report of Three Cases Observed since Birth. *Helv Paediatr Acta* **1983**, *38*, 383–398.
75. Jurca, C.M.; Iuhas, O.; Kozma, K.; Petchesi, C.D.; Zaha, D.C.; Bembea, M.; Jurca, S.; Paul, C.; Jurca, A.D. Effects of Burosumab Treatment on Two Siblings with X-Linked Hypophosphatemia. Case Report and Literature Review. *Genes (Basel)* **2022**, *13*, doi:10.3390/genes13081392.
76. Friedman, N.E.; Lobaugh, B.; Drezner, M.K. Effects of Calcitriol and Phosphorus Therapy on the Growth of Patients with X-Linked Hypophosphatemia. *J Clin Endocrinol Metab* **1993**, *76*, 839–844, doi:10.1210/jcem.76.4.8473393.
77. Ekpebegh, C.O.; Blanco-Blanco, E. Familial Hypophosphataemic Rickets Affecting a Father and His Two Daughters: A Case Report. *West Afr J Med* **2010**, *29*, 271–274.
78. Lubani, M.M.; Khuffash, F.A.; Reavey, P.C.; Sharda, D.C.; Alshab, T.S. Familial Hypophosphataemic Rickets: Experience with 24 Children from Kuwait. *Ann Trop Paediatr* **1990**, *10*, 377–381, doi:10.1080/02724936.1990.11747461.
79. Ring, E.; Zobel, G.; Riccabona, M. [Familial hypophosphatemia]. *Wien Klin Wochenschr* **1990**, *102*, 303–306.
80. Zhao, Y.; Yang, F.; Wang, L.; Che, H. Familial Hypophosphatemic Rickets Caused by a PHEX Gene Mutation Accompanied by a NPR2 Missense Mutation. *J Pediatr Endocrinol Metab* **2020**, *33*, 305–311, doi:10.1515/jpem-2019-0380.
81. Ziller, R. [Genu-varum and-valgum surgery in childhood]. *Beitr Orthop Traumatol* **1977**, *24*, 220–226.
82. McNair, S.L.; Stickler, G.B. Growth in Familial Hypophosphatemic Vitamin-D-Resistant Rickets. *N Engl J Med* **1969**, *281*, 512–516.
83. Bhadada, S.K.; Bhansali, A.; Upreti, V.; Dutta, P.; Santosh, R.; Das, S.; Nahar, U. Hypophosphataemic Rickets/Osteomalacia: A Descriptive Analysis. *Indian J Med Res* **2010**, *131*, 399–404.
84. Novais, E.; Stevens, P.M. Hypophosphatemic Rickets: The Role of Hemiepiphysiodesis. *J Pediatr Orthop* **2006**, *26*, 238–244, doi:10.1097/01.bpo.0000218531.66856.b7.
85. Yuan, L.; Wu, S.; Xu, H.; Xiao, J.; Yang, Z.; Xia, H.; Liu, A.; Hu, P.; Lu, A.; Chen, Y.; et al. Identification of a Novel PHEX Mutation in a Chinese Family with X-Linked Hypophosphatemic Rickets Using Exome Sequencing. *Biol Chem* **2015**, *396*, 27–33, doi:10.1515/hsz-2014-0187.
86. Cao, Y.; You, Y.; Wang, Q.; Ren, X.; Li, S.; Li, L.; Xia, W.; Guan, X.; Yang, T.; Ikegawa, S.; et al. Identification of Six Novel Variants from Nine Chinese Families with Hypophosphatemic Rickets. *BMC Med Genomics* **2022**, *15*, 161, doi:10.1186/s12920-022-01305-w.
87. Mindler, G.T.; Kranzl, A.; Stauffer, A.; Kocijan, R.; Ganger, R.; Radler, C.; Haeusler, G.; Raimann, A. Lower Limb Deformity and Gait Deviations Among Adolescents and Adults With X-Linked Hypophosphatemia. *Front Endocrinol (Lausanne)* **2021**, *12*, 754084, doi:10.3389/fendo.2021.754084.
88. Lempicki, M.; Rothenbuhler, A.; Merzoug, V.; Franchi-Abella, S.; Chaussain, C.; Adamsbaum, C.; Linglart, A. Magnetic Resonance Imaging Features as Surrogate Markers of X-Linked Hypophosphatemic Rickets Activity. *Horm Res Paediatr* **2017**, *87*, 244–253, doi:10.1159/000464142.
89. Fucentese, S.F.; Neuhaus, T.J.; Ramseier, L.E.; Ulrich Exner, G. Metabolic and Orthopedic Management of X-Linked Vitamin D-Resistant Hypophosphatemic Rickets. *J Child Orthop* **2008**, *2*, 285–291, doi:10.1007/s11832-008-0118-9.
90. Makitie, O.; Toiviainen-Salo, S.; Marttinen, E.; Kaitila, I.; Sochett, E.; Sipila, I. Metabolic Control and Growth during Exclusive Growth Hormone Treatment in X-Linked Hypophosphatemic Rickets. *Horm Res* **2008**, *69*, 212–220, doi:10.1159/000113021.

91. Rush, E.T.; Johnson, B.; Aradhya, S.; Beltran, D.; Bristow, S.L.; Eisenbeis, S.; Guerra, N.E.; Krolczyk, S.; Miller, N.; Morales, A.; et al. Molecular Diagnoses of X-Linked and Other Genetic Hypophosphatemias: Results From a Sponsored Genetic Testing Program. *J Bone Miner Res* **2022**, *37*, 202–214, doi:10.1002/jbmr.4454.
92. Uday, S.; Shaw, N.J.; Mughal, M.Z.; Randell, T.; Högler, W.; Santos, R.; Padidela, R. Monitoring Response to Conventional Treatment in Children with XLH: Value of ALP and Rickets Severity Score (RSS) in a Real World Setting. *Bone* **2021**, *151*, 116025, doi:10.1016/j.bone.2021.116025.
93. Şıklar, Z.; Turan, S.; Bereket, A.; Baş, F.; Güran, T.; Akberzade, A.; Abacı, A.; Demir, K.; Böber, E.; Özbek, M.N.; et al. Nationwide Turkish Cohort Study of Hypophosphatemic Rickets. *J Clin Res Pediatr Endocrinol* **2020**, *12*, 150–159, doi:10.4274/jcrpe.galenos.2019.2019.0098.
94. Huang, Y.; Mei, L.; Pan, Q.; Tan, H.; Quan, Y.; Gui, B.; Chang, J.; Ma, R.; Peng, Y.; Yang, P.; et al. Novel de Novo Nonsense Mutation of the PHEX Gene (p.Lys50Ter) in a Chinese Patient with Hypophosphatemic Rickets. *Gene* **2015**, *565*, 150–154, doi:10.1016/j.gene.2015.03.066.
95. Chou, Y.-Y.; Chao, S.-C.; Tsai, S.-C.; Lin, S.-J. Novel PHEX Gene Mutations in Two Taiwanese Patients with Hypophosphatemic Rickets. *J Formos Med Assoc* **2005**, *104*, 198–202.
96. Kienitz, T.; Ventz, M.; Kaminsky, E.; Quinkler, M. Novel PHEX Nonsense Mutation in a Patient with X-Linked Hypophosphatemic Rickets and Review of Current Therapeutic Regimens. *Exp Clin Endocrinol Diabetes* **2011**, *119*, 431–435, doi:10.1055/s-0031-1277162.
97. Eyres, K.S.; Brown, J.; Douglas, D.L. Osteotomy and Intramedullary Nailing for the Correction of Progressive Deformity in Vitamin D-Resistant Hypophosphataemic Rickets. *J R Coll Surg Edinb* **1993**, *38*, 50–54.
98. Gizard, A.; Rothenbuhler, A.; Pejin, Z.; Finidori, G.; Glorion, C.; de Billy, B.; Linglart, A.; Wicart, P. Outcomes of Orthopedic Surgery in a Cohort of 49 Patients with X-Linked Hypophosphatemic Rickets (XLHR). *Endocr Connect* **2017**, *6*, 566–573, doi:10.1530/EC-17-0154.
99. Mindler, G.T.; Stauffer, A.; Kranzl, A.; Penzkofer, S.; Ganger, R.; Radler, C.; Haeusler, G.; Raimann, A. Persistent Lower Limb Deformities Despite Amelioration of Rickets in X-Linked Hypophosphatemia (XLH)—A Prospective Observational Study. *Front. Endocrinol.* **2022**, *13*, 866170. https://doi.org/10.3389/fendo.2022.866170.
100. Grote, C.W.; Nepple, J.J.; Schoenecker, P.L.; Gottesman, G.S.; Gordon, J.E.; Miller, M.L. Predicting Rates of Angular Correction After Hemiepiphysiodesis in Patients With X-Linked Hypophosphatemic Rickets. *J Pediatr Orthop* **2023**, *43*, 379–385, doi:10.1097/BPO.0000000000002393.
101. Sandy, J.L.; Nunez, C.; Wheeler, B.J.; Jefferies, C.; Morris, A.; Siafarikas, A.; Rodda, C.P.; Simm, P.; Biggin, A.; Aum, S.; et al. Prevalence and Characteristics of Paediatric X-Linked Hypophosphataemia in Australia and New Zealand: Results from the Australian and the New Zealand Paediatric Surveillance Units Survey. *Bone* **2023**, *173*, 116791, doi:10.1016/j.bone.2023.116791.
102. Moreira, C.A.; Costa, T.M.R.L.; Marques, J.V.O.; Sylvestre, L.; Almeida, A.C.R.; Maluf, E.M.C.P.; Borba, V.Z.C. Prevalence and Clinical Characteristics of X-Linked Hypophosphatemia in Paraná, Southern Brazil. *Arch Endocrinol Metab* **2021**, *64*, 796–802, doi:10.20945/2359-3997000000296.
103. Baroncelli, G.I.; Angiolini, M.; Ninni, E.; Galli, V.; Saggese, R.; Giuca, M.R. Prevalence and Pathogenesis of Dental and Periodontal Lesions in Children with X-Linked Hypophosphatemic Rickets. *Eur J Paediatr Dent* **2006**, *7*, 61–66.
104. Evans, G.A.; Arulanantham, K.; Gage, J.R. Primary Hypophosphatemic Rickets. Effect of Oral Phosphate and Vitamin D on Growth and Surgical Treatment. *J Bone Joint Surg Am* **1980**, *62*, 1130–1138.
105. Rubinovitch, M.; Said, S.E.; Glorieux, F.H.; Cruess, R.L.; Rogala, E. Principles and Results of Corrective Lower Limb Osteotomies for Patients with Vitamin D-Resistant Hypophosphatemic Rickets. *Clin Orthop Relat Res* **1988**, 264–270.
106. Bonnet-Lebrun, A.; Linglart, A.; De Tienda, M.; Ouchrif, Y.; Berkenou, J.; Assi, A.; Wicart, P.; Skalli, W. Quantitative Analysis of Lower Limb and Pelvic Deformities in Children with X-Linked Hypophosphatemic Rickets. *Orthop. Traumatol. Surg. Res.* **2023**, *109*, 103187. https://doi.org/10.1016/j.otsr.2021.103187.

107. Aiello, F.; Pasquali, D.; Baronio, F.; Cassio, A.; Rossi, C.; Di Fraia, R.; Carotenuto, R.; Digitale, L.; Festa, A.; Luongo, C.; et al. Rare PHEX Intron Variant Causes Complete and Severe Phenotype in a Family with Hypophosphatemic Rickets: A Case Report. *J Pediatr Endocrinol Metab* **2023**, *36*, 91–95, doi:10.1515/jpem-2022-0365.
108. Popkov, A.; Aranovich, A.; Popkov, D. Results of Deformity Correction in Children with X-Linked Hereditary Hypophosphatemic Rickets by External Fixation or Combined Technique. *Int Orthop* **2015**, *39*, 2423–2431, doi:10.1007/s00264-015-2814-7.
109. Greene, W.B.; Kahler, S.G. Surgical Aspects of Limb Deformity in Hypophosphatemic Rickets. *South Med J* **1985**, *78*, 1185–1189, doi:10.1097/00007611-198510000-00011.
110. Feng, W.-J.; Dai, Z.-Z.; Xiong, Q.-G.; Wu, Z.-K. Temporary Hemiepiphysiodesis Using Eight-Plates for Angular Deformities of the Lower Extremities in Children with X-Linked Hypophosphataemic Rickets. *Int Orthop* **2023**, *47*, 763–771, doi:10.1007/s00264-023-05688-y.
111. Loeffler, R.D.J.; Sherman, F.C. The Effect of Treatment on Growth and Deformity in Hypophosphatemic Vitamin D-Resistant Rickets. *Clin Orthop Relat Res* **1982**, 4–10.
112. Horn, A.; Wright, J.; Bockenhauer, D.; Van't Hoff, W.; Eastwood, D.M. The Orthopaedic Management of Lower Limb Deformity in Hypophosphataemic Rickets. *J Child Orthop* **2017**, *11*, 298–305, doi:10.1302/1863-2548.11.170003.
113. Song, S.-H.; Lee, H.; Jeong, J.-M.; Cho, W.-I.; Kim, S.E.; Song, H.-R. The Significance of Serum Phosphate Level on Healing Index and Its Relative Effects in Skeletally Immature and Mature Patients with Hypophosphatemic Rickets. *Biomed. Res. Int.* **2014**, *2014*, 569530. https://doi.org/10.1155/2014/569530.
114. Zagari, M.C.; Chiarello, P.; Iuliano, S.; D'Antona, L.; Rocca, V.; Colao, E.; Perrotti, N.; Greco, F.; Iuliano, R.; Aversa, A. The Variant p.Ala84Pro Is Causative of X-Linked Hypophosphatemic Rickets: Possible Relationship with Burosumab Swinging Response in Adults. *Genes (Basel)* **2022**, *14*, doi:10.3390/genes14010080.
115. McAlister, W.H.; Kim, G.S.; Whyte, M.P. Tibial Bowing Exacerbated by Partial Premature Epiphyseal Closure in Sex-Linked Hypophosphatemic Rickets. *Radiology* **1987**, *162*, 461–463, doi:10.1148/radiology.162.2.3025921.
116. Eguchi, M.; Kaibara, N. Treatment of Hypophosphataemic Vitamin D-Resistant Rickets and Adult Presenting Hypophosphataemic Vitamin D-Resistant Osteomalacia. *Int Orthop* **1980**, *3*, 257–264, doi:10.1007/BF00266019.
117. Kanel, J.S.; Price, C.T. Unilateral External Fixation for Corrective Osteotomies in Patients with Hypophosphatemic Rickets. *J Pediatr Orthop* **1995**, *15*, 232–235.
118. LITMAN, N.N.; ULSTROM, R.A.; WESTIN, W.W. Vitamin D Resistant Rickets. *Calif Med* **1957**, *86*, 248–253.
119. Godfrey, E.K.; Mussa, F.; Kazahura, P.; Shoo, A.; Naburi, H.; Manji, K.P. Vitamin D-Resistant Rickets Diagnostics and Treatment Challenges at Muhimbili National Hospital, Tanzania. *Case Rep Endocrinol* **2020**, *2020*, 1547170, doi:10.1155/2020/1547170.
120. Tavana, N.; Ting, T.H.; Lai, K.; Kennerson, M.L.; Thilakavathy, K. Whole Exome Sequencing Identifies Two Novel Variants in PHEX and DMP1 in Malaysian Children with Hypophosphatemic Rickets. *Ital J Pediatr* **2022**, *48*, 193, doi:10.1186/s13052-022-01385-5.
121. Al Kaissi, A.; Farr, S.; Ganger, R.; Klaushofer, K.; Grill, F. Windswept Lower Limb Deformities in Patients with Hypophosphataemic Rickets. *Swiss Med Wkly* **2013**, *143*, w13904, doi:10.4414/smw.2013.13904.
122. Dahir, K.M.; Black, M.; Gottesman, G.S.; Imel, E.A.; Mumm, S.; Nichols, C.M.; Whyte, M.P. X-Linked Hypophosphatemia Caused by the Prevailing North American PHEX Variant c.*231A>G; Exon 13-15 Duplication Is Often Misdiagnosed as Ankylosing Spondylitis and Manifests in Both Men and Women. *JBMR Plus* **2022**, *6*, e10692, doi:10.1002/jbm4.10692.
123. Reid, I.R.; Hardy, D.C.; Murphy, W.A.; Teitelbaum, S.L.; Bergfeld, M.A.; Whyte, M.P. X-Linked Hypophosphatemia: A Clinical, Biochemical, and Histopathologic Assessment of Morbidity in Adults. *Medicine (Baltimore)* **1989**, *68*, 336–352.
124. Smith, P.S.; Gottesman, G.S.; Zhang, F.; Cook, F.; Ramirez, B.; Wenkert, D.; Wollberg, V.; Huskey, M.; Mumm, S.; Whyte, M.P. X-Linked Hypophosphatemia: Uniquely Mild Disease Associated With PHEX 3'-

UTR Mutation c.*231A>G (A Retrospective Case-Control Study). *J Bone Miner Res* **2020**, *35*, 920–931, doi:10.1002/jbmr.3955.

125. Emma, F.; Cappa, M.; Antoniazzi, F.; Bianchi, M.L.; Chiodini, I.; Eller Vainicher, C.; Di Iorgi, N.; Maghnie, M.; Cassio, A.; Balsamo, A.; et al. X-Linked Hypophosphatemic Rickets: An Italian Experts' Opinion Survey. *Ital J Pediatr* **2019**, *45*, 67, doi:10.1186/s13052-019-0654-6.
126. Nielsen, L.H.; Rahbek, E.T.; Beck-Nielsen, S.S.; Christesen, H.T. Treatment of Hypophosphataemic Rickets in Children Remains a Challenge. *Dan Med J* **2014**, *61*, A4874.
